# Clinical Overview of Progressive Fibrotic Interstitial Lung Disease

**DOI:** 10.3389/fmed.2022.858339

**Published:** 2022-03-15

**Authors:** Amy Hajari Case

**Affiliations:** Piedmont Atlanta Hospital, Atlanta, GA, United States

**Keywords:** pulmonary fibrosis, interstitial lung disease (ILD), connective tissue disease-associated ILD, hypersensitivity pneumonitis (HP), antifibrotic

## Abstract

Interstitial lung diseases (ILD) on the whole have variable prognoses, but there are those which manifest with fibrosis and are characterized by disease progression. Chief among these is idiopathic pulmonary fibrosis, but other ILDs, including autoimmune ILD and chronic hypersensitivity pneumonitis, may have a progressive fibrotic phenotype also. A usual interstitial pneumonia pattern of lung involvement is a prominent risk factor for such a course, suggesting shared fibrotic pathways that may be targeted by antifibrotic therapies. This brief review describes ILDs that are most commonly fibrotic, shared risk factors for development of PF-ILD, and evidence for antifibrotic use in their management.

## Introduction

Interstitial lung diseases (ILD) are a group of diffuse parenchymal lung diseases that cause inflammation, fibrosis, or both. Over 200 clinical diagnoses comprise this group of lung diseases, including those categorized as idiopathic interstitial pneumonias (IIP), exposure-related diseases such as hypersensitivity pneumonitis (HP), and connective tissue disease-related (CTD) ILDs, among others. Of these, a subset may manifest with pulmonary fibrosis and can demonstrate a progressive phenotype (1, 2). Though not formally defined, features that suggest progressive fibrosing ILD (PF-ILD) include decline in lung function as measured by forced vital capacity (FVC) or diffusing capacity of the lung for carbon monoxide (DLCO), radiographic progression of fibrotic features on high resolution computed tomography (HRCT), or worsening symptoms despite treatment (3).

The epidemiology of IPF is well-described with incidence ranging from 3 to 9 cases per 100,000 people per year in Europe and North America (4, 5) with incidence increasing over time (4). One review of Medicare data reported incidence of IPF to be as high as 93.7 per 100,000 people per year for those over age 65 (6).

The retrospective PROGRESS study sought to characterize PF-ILD other than IPF in a large, single center cohort in France (7). Among 1,395 patients, 617 had non-IPF fibrosing ILD, and 27% of these experienced disease progression, the most common etiologies being CTD-ILD (46%), unclassifiable ILD (25%), IIP (15%), and chronic HP (7%) (7).

## ILDs at Risk For a Progressive Fibrosing Phenotype

### Idiopathic Interstitial Pneumonias

Multiple specific ILDs may manifest with a progressive fibrosing phenotype. The archetypical disease manifesting in this way is idiopathic pulmonary fibrosis (IPF), the most common of the IIPs (8). IPF is characterized by a pattern of usual interstitial pneumonia (UIP) on HRCT or histopathology (8), not associated with identifiable etiology, and almost invariably progressive in nature. The development of fibrosis follows recurrent injury to the lung epithelium with aberrant healing and collagen deposition by myofibroblasts (9). Risk factors for disease progression include increasing age (10), oxygen use at rest (10), lower or decline in FVC (10, 11), and lower DLCO (10), and the clinical course can be complicated by episodes of acute respiratory deterioration or acute exacerbations (12).

IIPs comprise a group of diagnoses defined primarily by their radiographic and pathologic features. While non-specific interstitial pneumonia (NSIP) is often a manifestation of lung injury related to autoimmune disease or drug toxicity, idiopathic NSIP is a distinct clinical entity with variable prognosis, a subset of cases having a progressive fibrotic phenotype (13). Radiographically, NSIP is characterized by bilateral ground glass opacities, fibrotic features of reticulation and traction bronchiectasis, and minimal honeycombing (13). Histological findings are predominantly fibrotic without honeycombing but can rarely manifest as uniformly cellular (13). Of those patients with an IIP, up to 15% can remain unclassifiable after diagnostic evaluation and multidisciplinary discussion due to overlapping features or discrepancy between clinical, radiographic, and pathologic findings (14, 15). Mortality rates for unclassifiable ILD appear better than those of IPF but worse than other non-IPF ILDs (14).

### Connective Tissue Disease-Related ILD

In CTD, ILD is mediated by systemic inflammation in the setting of autoimmunity. Autoimmune diagnoses associated with ILD include systemic sclerosis (SScl), rheumatoid arthritis (RA), myositis syndromes, systemic lupus erythematosus, Sjogren's syndrome, and mixed CTD. Clinical evaluation for evidence of systemic autoimmune disease is part of the diagnostic process for ILD (8). NSIP is the most common lung injury pattern in CTD-ILD, and organizing pneumonia (OP) and lymphocytic interstitial pneumonia (LIP) are also seen (16). CTD-ILD has a more favorable prognosis than IPF and other IIPs, regardless of pattern (17), but common risk factors for progression and poor outcomes across primary diagnoses include UIP pattern, lower baseline FVC and DLCO, worsening pulmonary function parameters over time, and a diagnosis of SScl vs. RA (18–31).

Though it is primarily characterized by skin and soft tissue involvement, pulmonary disease is a frequent and sometimes only manifestation of SScl (32). ILD is a significant contributor to morbidity and mortality in SScl. In the EULAR Scleroderma Trials and Research cohort, pulmonary fibrosis caused 35% of SScl-related deaths (33). Male sex, African-American race, positive anti-Scl-70 antibody, diffuse type of SScl, and digital ulcers are associated with development of ILD (32, 34, 35). Unlike other CTD-ILDs, treatment regimens for SScl-ILD have been studied in prospective clinical trials. Immunomodulators mycophenolate mofetil (MMF), cyclophosphamide, azathioprine, and tocilizumab as well as the antifibrotic agent, nintedanib, have all shown benefit in placebo-controlled studies.

RA is defined primarily by inflammatory arthritis but with frequent extraarticular involvement. The lung is a common site of extra-articular disease, with radiographic changes consistent with ILD in as many as 2/3 of patients (36, 37). Risk factors for development of ILD include male sex, cigarette smoking, high titer rheumatoid factor (RF) and anti-citric citrullinated peptide (CCP) levels, advanced age, severity of articular disease, and presence of the MUC5B promotor variant (37–40). Unlike other CTDs, in RA, UIP is the most frequently reported pattern of lung involvement with NSIP, OP, desquamative interstitial pneumonia, LIP, and diffuse alveolar damage also reported (28, 29, 37, 39, 41). Disease progression is not universal. While the mainstay of therapy for RA-ILD generally includes corticosteroid therapy with or without a cytotoxic agent, prospective trials are lacking. Reports of immunomodulator use in RA-ILD to date have not focused specifically on progressive fibrotic disease (37, 42–44).

In some cases of ILD, there are features of underlying CTD that do not fulfill the criteria for a specific autoimmune diagnosis. Further, ILD can be the sole or initial presenting manifestation of CTD (45, 46). The term “interstitial pneumonia with autoimmune features” (IPAF) has been proposed to describe “lung-dominant” CTD that cannot be classified as another specific entity (16). Proposed criteria include features in clinical, serologic, and morphologic domains (16). In a retrospective cohort of ILD patients, patients meeting criteria for IPAF had worse survival than those with other CTD-ILD (31).

### Hypersensitivity Pneumonitis

HP is an immune-mediated response to inhaled environmental antigens causing ILD in susceptible individuals. Recent guidelines separate HP in to non-fibrotic and fibrotic forms (47, 48), the latter also termed “chronic HP.” In the diagnosis of HP identification of an inciting antigen significantly influences the pre-test probability of the disease, but is not necessary to the diagnosis (47–49). Antigen avoidance is a key aspect of management, however about half of patients with chronic HP do not have identifiable inciting antigen, which is associated with worse prognosis (50). Other clinical factors associated with worse prognosis include older age, male sex, smoking history, lower baseline FVC or DLCO, and absent lymphocytosis on bronchoalveolar lavage (47, 51). In addition to antigen avoidance, corticosteroids and steroid-sparing agents are often used, with limited evidence (52).

### Sarcoidosis

Sarcoidosis is a disease of unknown etiology, which can affect any organ system and is characterized by non-necrotizing granulomatous inflammation. The lungs are most commonly affected, and while two-thirds of patients experience spontaneous remission, 10–20% can go on to develop pulmonary fibrosis (53), and respiratory failure is the most common cause of death (54–56). The development of fibrosis is thought to be related to ongoing granulomatous inflammation, but risk factors for progressive pulmonary fibrosis are not well-characterized (57, 58). When active granulomatous inflammation is present, corticosteroids are used to improve function and symptoms, and steroid-sparing agents are employed to reduce the corticosteroid dose and toxicity (59).

### Antifibrotics in Progressive Fibrotic ILD

Given the morbidity and mortality implications of progressive PF in all types of ILD and the limitations of existing treatment strategies, primarily immunomodulatory agents, there is interest in utilizing antifibrotic agents in these conditions. The antifibrotic drugs, nintedanib and pirfenidone, both have proven clinical benefit in IPF (60, 61), and both are being studied in non-IPF PF-ILD.

Two recent studies, demonstrated efficacy of nintedanib at slowing disease progression in non-IPF ILD (62, 63). In the SCENSCIS trial of SScl-ILD involving at least 10% of the lungs on HRCT, nintedanib lowered annual rate of FVC decline compared with placebo (−52.4 ml vs. −93.3, *p* = 0.04) with 48% of subjects receiving mycophenolate at time of randomization (62). The INBUILD trial demonstrated similar efficacy in a broader cohort of PF-ILD subjects with the nintedanib group demonstrating lower annual rate of FVC decline vs. placebo (−80.8 ml vs. −187.8 ml, *p* < 0.001), a reduction that was more pronounced in the subgroup with UIP-like fibrotic features on HRCT (63). A subsequent subgroup analysis of the INBUILD cohort demonstrated this effect over multiple specific ILD diagnoses (2).

Phase 2 studies of pirfenidone in non-IPF PF-ILD of multiple etiologies and unclassifiable PF-ILD both demonstrated attenuated FVC decline in treated subjects (64, 65). The open-label LOTUSS trial demonstrated tolerability and safety of pirfenidone when used with or without MMF in SScl-ILD (66), and the Scleroderma Lung Study III using pirfenidone in SScl-ILD is ongoing (NCT03221257). Additional investigations of pirfenidone are underway in RA-ILD (NCT02808871), chronic HP (NCT02958917, NCT02496182), and fibrotic sarcoidosis (NCT03260556).

A number of novel therapies are currently in development for IPF, and if effective, it is anticipated that additional study in non-IPF PF-ILDs would be undertaken. Specific to PF-ILD, the LPA_1_ antagonist, BMS-986278, has shown promise in pre-clinical and phase I studies (67, 68) and is currently in phase 2 clinical trials, with study arms for both IPF and PF-ILD subjects (NCT0438681).

## Conclusion

While progress has been made in the treatment of IPF with the availability of 2 novel antifibrotic therapies and multiple other therapies in development, there remain limitations in both understanding and management in other types of ILD, which can portend a poor prognosis when progressive. Accurate diagnosis remains vital to management, but shared fibrosis pathways appear to confer a favorable respond to antifibrotic treatments across multiple etiologies.

## Author Contributions

The author confirms being the sole contributor of this work and has approved it for publication.

## Conflict of Interest

AC serves as a site principal investigator for clinical trials sponsored by Boehringer-Ingelheim, Genentech/Roche, and Bristol-Myers-Squibb.

## Publisher's Note

All claims expressed in this article are solely those of the authors and do not necessarily represent those of their affiliated organizations, or those of the publisher, the editors and the reviewers. Any product that may be evaluated in this article, or claim that may be made by its manufacturer, is not guaranteed or endorsed by the publisher.
